# Complications analysis of posterior vertebral column resection in 40 patients with spinal tumors

**DOI:** 10.3892/etm.2014.1929

**Published:** 2014-08-22

**Authors:** YU FAN, YU XIA, HONG ZHAO, JIANGUO ZHANG, SHUGANG LI, YE TIAN, XISHENG WENG, GUIXING QIU

**Affiliations:** 1 Department of Orthopaedics, Peking Union Medical College Hospital, Peking Union Medical College, Beijing 100730, P.R. China; 2 Department of Pathology, Peking Union Medical College Hospital, Peking Union Medical College, Beijing 100730, P.R. China

**Keywords:** vertebral column resection, spinal tumor, complication

## Abstract

The aim of the present study was to summarize and analyze the complications of posterior vertebral column resection in patients with spinal tumors. The complications of 40 patients following surgery were recorded, and surgery-related parameters including segments, bleeding volume and surgical duration were recorded and analyzed. SPSS 12.0 software was used to analyze the correlation between the complications and these parameters retrospectively. A total of 36 complications were reported. The median follow-up duration of the patients was 14 months (range, 4–78 months). Transient late tracheal extubation was associated with higher intraoperative bleeding volume, lower preoperative forced vital capacity and forced expiratory volume in 1 sec. Replaced spinal segment subsidence was associated with increased duration of surgery, higher intraoperative bleeding volume and higher total blood transfusion volume. Thrombocytopenia was associated with increased duration of surgery and higher total blood transfusion volume. The majority of the complications were minor and did not affect the recovery of the patients. Active prevention is necessary to reduce the incidence of complications, in particular, major ones.

## Introduction

Bone metastases are a frequent problem in patients affected by cancer, and the oncological treatment of these tumors includes surgery, radiotherapy and chemotherapy. The spine is the most common site of tumor metastasis, and metastatic tumors of the spine are a common type of malignant spinal tumors. In total, 12–20% of patients with malignant tumor initially showed characteristics of spinal metastasis (1). In particular, surgery is indicated in cases of spinal instability, cord compression, failure of previous radiotherapy and when the diagnosis is in doubt. In recent years, with the development of operation and spinal reconstruction technology, the operation treatment of vertebral metastases strategy emphasize that based on the specific metastasis position, nerve compression and decompression should be directly performed in these positions, and should perform spinal stability reconstruction (1). Posterior vertebral column resection is an effective technique that has been widely applied to treat severe rigid spinal deformities and tumors (2–4). The surgery is able to radically resect the regional lesions; however, it requires complex skills, and the associated complications should not be neglected. The majority of previous studies (5–7) have focused on the efficacy of this surgery, omitting any systemic review of the complications. The present study summarizes the complications of 40 consecutive cases of posterior vertebral column resection and systematically analyses the complications of this procedure in 40 patients with spinal tumors.

## Patients and methods

### Patient characteristics

Patients who underwent routine vertebral column resection were included in this retrospective study. The inclusion criteria were as follows: Patients were aged ≥18 years and had a diagnosis of primary, secondary or metastatic spinal tumor. The exclusion criteria were patients who: i) were not surgical candidates for spine tumor removal, as determined by the surgical team; ii) had undergone previous spinal surgery for tumor removal; iii) in the opinion of the investigator may not have been able to comply with the safety monitoring requirements of the study.

The study was approved by the ethics committee of the Peking Union Medical College Hospital (Beijing, China). The ethics committee approved the associated treatment, data collection and follow-up of these patients. Written informed consent was obtained from all the subjects between August 2005 and December 2011.

### Surgical procedure

Briefly, following general anesthesia, the patients were placed in a prone position. A mid-line linear incision was created and the ligamentous attachments and muscle were dissected to the tips of the transverse process over the levels of decompression and posterior fixation. During the process, great care was taken to avoid the transmission of pressure to the spinal cord.

In the thoracic spine, a bilateral costotransversectomy of 5–6 cm of the medial ribs associated with the resection level was performed. Resections were later performed either piecemeal or en bloc. The pleura were protected carefully. The dura was cautiously elevated posteriorly, and the epidural veins were pre-cauterized by bipolar cautery. The lateral cortex of the vertebra was then exposed by blunt dissection. A pre-contoured rod was connected to the screws on the opposite side to stabilize the spine prior to removing the vertebral body. Osteotomies were used to remove the vertebral body along the cartilage endplate. A bone knife or wire saw was used to cut off the vertebral pedicle. The upper and lower discs, including the cartilage endplate, were completely removed until the resected bone was exposed, and the other side of the vertebra was then removed using the same procedure. Subsequently, anterior reconstruction with a titanium mesh cage or artificial vertebral body was used for vertebral body replacement. Posterolateral fusion was performed, and during the surgical process, controlled hypotension was used and the vertebra was resected as quickly as possible to minimise blood loss.

### Postoperative management

Antibiotics were infused intravenously between the first and third postoperative days (the duration was normally limited to 48 h but was prolonged to six days in one case due to pneumonia). The drainage was removed within 24–48 h after the surgery. The patients were allowed to sit up and gradually walk three days after the surgery with a brace. If the patient recovered well, the brace was removed 24 weeks after the surgery.

### Outcome assessment

The intra- and postoperative complications, resected segment, duration of surgery, intraoperative blood loss, transfusion volume, serum levels of hemoglobin and platelets between post- and preoperation, preoperational pulmonary function test results, postoperative liver and renal function, and timing of extubation were recorded. Estimated blood loss was recorded at the end of the surgery by a single staff member from the anesthesia department. An X-ray was applied to evaluate the subsidence and excursion of the replaced spinal segment. In the present study the complications were classified as major or minor (8). Major complications were those that may affect or change the expected recovery, and the others were designated as minor complications. The complications were classified as intraoperative and early and late postoperative. Early complications were those occurring within the first 30 days after surgery, and late complications were those occurring after 30 days. These three types of complications were further classified as major or minor.

### Statistical analysis

Normally distributed continuous data are presented as the mean ± standard deviation and were compared using Student’s t-tests. Non-normally distributed continuous data were compared using the Mann-Whitney U test. Qualitative data are expressed as frequencies and percentages. The Fisher’s exact or χ^2^ tests were used to examine the correlations between the qualitative variables. Differences were considered statistically significant when P<0.05. SPSS software version 12.0 (SPSS, Inc., Chicago, IL, USA) was used to conduct the data analysis.

## Results

### Patient information

**Figure 1:**
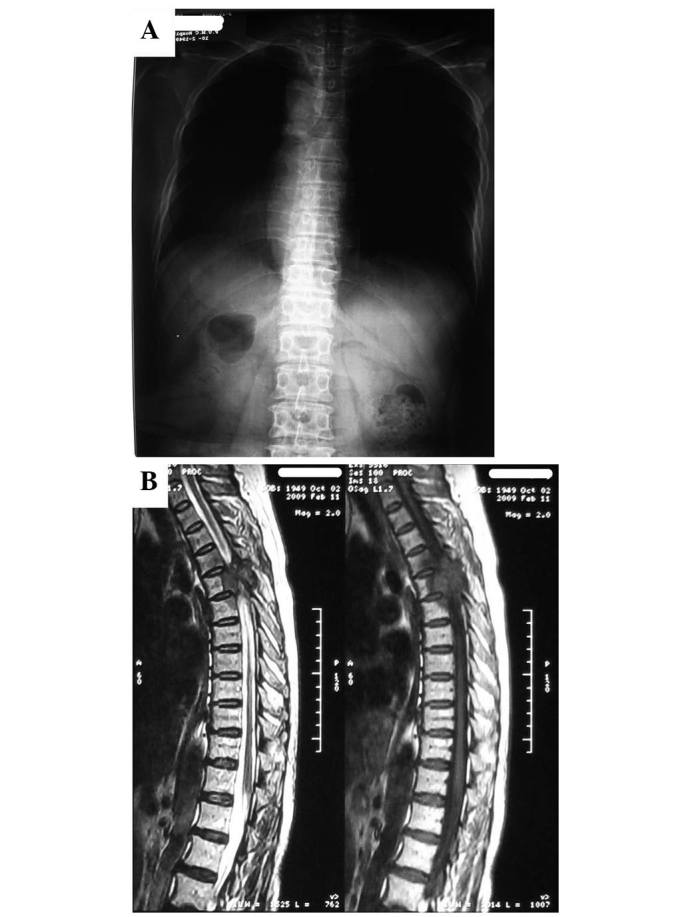
Image examination result of a tumor in the spine. (A) Lateral X-Ray of the thoracic spine of a patient with a thoracic vertebral metastatic tumor (lung cancer), with the lesion located at T4 and T5; (B) magnetic resonance imaging of the thoracic spine showed the vertebral metastatic tumor.

A total of 22 males and 18 females were selected for inclusion in the present study. The median age of these patients was 52 years (range, 20–78 years). Primary bone tumors accounted for 13 cases (32.5%) and metastatic lesions in bone for 27 cases (67.5%). A total of 40 tumors were detected through X-ray or magnetic resonance imaging examination. Representative images are shown in Fig. 1. It was observed that 10 tumors were located at the lumbar vertebrae and the remaining 30 tumors were located at the thoracic vertebrae.

Two adjacent thoracic vertebrae were resected in four patients, and single vertebral resection was performed in the other patients. No cases of mortality occurred, but two cases survived for <6 months. The neurological function of the patients was evaluated pre- and postoperatively using the five-tier Frankel grading scale (9). One patient exhibited complete motor and sensory loss (Frankel Grade A); two had complete motor loss with some sensation preserved (Frankel Grade B); 22 had an incomplete loss of motor function (including three patients of Frankel Grade C and 19 of Frankel Grade D) and 15 retained normal motor and sensory functions (Frankel Grade E).

### Duration of surgery, blood transfusion and follow-up period

The ranges and medians of these parameters were as follows: Duration of surgery, range 135–470 min and mean±standard deviation, 305.8±78.2 min; blood loss volume, range 600–11,000 ml, median 2,400 ml; blood transfusion volume, range 0–26,200 ml, median 2,600 ml; and follow-up time, range 4–78 months, median 14 months.

### Intraoperative and early and late postoperative complications

**Figure 2:**
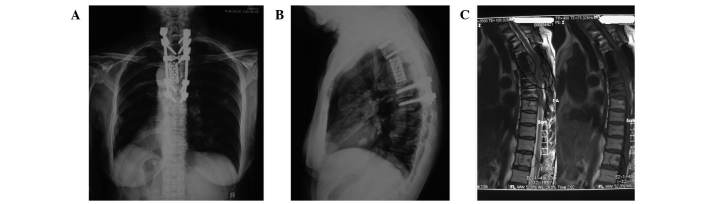
Image examination results of a patient two years after posterior vertebral column resection. (A) Anterior-posterior and (B) lateral X-rays of the thoracic spine two years after surgery; (C) magnetic resonance imaging of the thoracic spine two years after surgery showed local recurrence and a new lesion at T12 (original lesion located at T4 and T5).

A total of two major and 34 minor complications occurred (Table I). The intraoperative and early and late postoperative complications included: two cases of intraoperative cerebral-spinal leakage; 10 of transient thrombocytopenia; one, acute renal failure and liver dysfunction; one of drainage tube retention due to allergy to the allograft bone with the allergy being controlled following the administration of anti-allergic drugs; one of infective shock due to pneumonia (a major complication); five of late tracheal extubation; two of hemothorax; one of pneumothorax; one of acute enteritis; one of transient cardiac ischemia following surgery; six of hardware subsidence; one of regional recurrence (a major complication, Fig. 2); and two patients who expected to ambulate normally following surgery but the Frankel grade was still C postoperatively, thus they felt dissatisfied with the limited motor function improvement.

### Late tracheal extubation is associated with higher intraoperative bleeding volume, and lower preoperative forced vital capacity (FVC) and forced expiratory volume in 1 sec (FEV1%)

**Figure 3:**
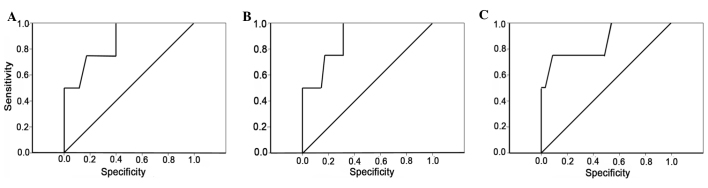
Receiver operating characteristic curves of intraoperative bleeding volume, FVC and FEV1% for late tracheal extubation. The highest diagnostic values and clinical significance were as follows: (A) When the blood loss was >4,100 ml, sensitivity and specificity were 75 and 82.9.2%, respectively; (B) when the preoperative FVC was <77.45, sensitivity and specificity were 100 and 68.6%, respectively. (C) when the preoperative FEV1% was <63.4, sensitivity and specificity were 75 and 91.4%, respectively. FVC, forced vital capacity; FEV1%, forced expiratory volume in 1 sec.

The parameters that could result in late tracheal extubation following surgery were analyzed. The Mann-Whitney U test indicated that transient late tracheal extubation was associated with a higher intraoperative bleeding volume (Z=−2.367, P=0.018; Table II). The Student’s t-test indicated that late tracheal extubation was associated with lower preoperative FVC (t=2.864, P=0.007) and FEV1% (t=2.563, P=0.015; Table III). A receiver operating characteristic (ROC) curve was drawn to analyze the correlation of late tracheal extubation with blood loss and preoperative FVC and FEV1% (Fig. 3).

### Replaced spinal segment subsidence is associated with longer duration of surgery

The Mann-Whitney U test indicated that hardware subsidence was associated with increased duration of surgery (Z=−2.158, P=0.031), intraoperative bleeding volume (Z=−2.895, P=0.004) and total blood transfusion volume (Z=−3.295, P=0.001, Table IV); and that thrombocytopenia was associated with increased operation duration (Z=−2.719, P=0.007) and total blood transfusion volume (Z=−2.273, P=0.023, Table V). The other factors were not statistically associated with late tracheal extubation, hardware subsidence and transient thrombocytopenia.

## Discussion

Previous studies have demonstrated that the general incidence of complications of spinal surgery is relatively high (10–12). The posterior vertebral column resection for spinal tumors is a widely used approach. The surgery requires the performing of extralesional or en bloc resection; therefore, additional anatomical structures may be resected (13), including the thoracic pleura, dura, muscle, nerve root and vessels. The resection may lead to a higher incidence of complications. En bloc resection may decrease tumor spread and recurrence; however, it is highly skillful and time-consuming, requiring the separation of the lateral and front sides of the vertebral body, bisection of the intervertebral disc and removal of the vertebrae surrounding the spinal cord. This surgery takes time to avoid causing injuries to the blood vessels and nerve roots; however, the incidence of nerve root stretching is high. Piecemeal resection removes the lesion gradually, and the individual performing the surgery is able to observe the front of the vertebra directly (14); therefore, the incidence of injuries of the vessels and nerves is relatively low. Since en bloc resection is highly challenging and risky, it should be performed with caution in appropriate patients, and certain associated factors should be considered, including the general condition, Tomita score, Weinstein-Boriani-Biagini stage, metastatic lesions and survival time. If the patient is not able to tolerate vertebral column resection, or the life expectancy is less than half a year, posterior or anterior decompression is a preferable option.

One case of pneumonia-induced shock occurred in the present study, in which the patient was old, exhibited poor general condition, had a long time of bed rest and were unable to expectorate. The shock was induced by pneumonia and was finally cured following active treatment. This indicates that it is necessary to avoid and manage pulmonary complications particularly for elderly patients who are weak and require a long period of bed rest (15).

FVC was used to evaluate the pulmonary volume and airway resistance. The FEV1% represents the proportion of the vital capacity that an individual is able to expire in the first second of expiration. In clinical practice, obstruction is defined as an FEV1% <70%. Although no chronic obstructive pulmonary disease or asthma was diagnosed in the present study, poor pulmonary function may decrease the compensation ability of the lungs and cause late tracheal extubation.

In the present study, the blood loss associated with the vertebral column resection was relatively high. This may have affected the hemodynamic stability. The statistical analysis revealed that late tracheal extubation was associated with the increase of blood loss, which indicated that an excessive discharge of blood could affect the oxygen supply of the patients. There were four cases of late tracheal extubation with a 2 h delay following surgery and one case with a 24 h delay, for whom the blood loss volume reached 11,000 ml. In addition, the ROC curve indicated that when the blood loss was >4,100 ml, the blood oxygen level should be monitored, which may lead to late tracheal extubation.

In addition, transient thrombocytopenia was found in 10 patients in this study, and the statistical analysis indicated that this was associated with an increased duration of surgery and total transfusion volume. No explanation for this connection has been found in the literature; however, one conclusion is that this may be caused by blood dilution following the application of a large amount of colloidal solution during the long surgery. Higher total transfusion volume also indicates that these patients required greater amounts of colloidal solution. Following the transfusion of red blood cells and plasma, the platelet count is likely to recover within three days, which should not affect the prognosis.

Hemodynamic disturbances may lead to transient ischemia of the vital organs. Thus, it may be speculated that the following complications were due to this cause: one case of cardiac ischemia, one case of liver dysfunction and one case of renal dysfunction. Since the renal dysfunction existed prior to the surgery, it was difficult to clarify the correlation with blood loss. Due to the limited number of samples, statistical analysis was not available. In addition, the blood loss of giant cell carcinomas was significantly higher than that of other tumors, indicating that the type of tumor may be associated with the level of blood loss.

Careful intraoperative manipulations and good protection of the surrounding tissues could avoid life-threatening complications, including inferior vena cava injuries (16). Two cases of cerebrospinal leakage occurred in the present study, and instant repair was performed. The drainage tube was subsequently removed 72 h after the surgery and the wound healed well without infection. Boriani *et al* (16) reported that delayed aortic dissection may be caused by the severe adherence of the aorta with the tumor. However, timely treatment, such as with aortic bypass surgery, may prevent this complication. Orthopedists should therefore be aware of the possible severe complications induced by intraoperative injuries of the surrounding tissues and ensure that they treat these problems in a timely manner.

The incidence of hemothorax is relatively lower in posterior compared with anterior surgeries. Hemothorax may be caused by a number of factors (17), including the inappropriate positioning of hooks and screws, which may penetrate the lungs and blood vessels, or the ribs penetrating the lungs and blood vessels during thoracoplasty, or occasionally the central venous catheterization may also penetrate the lungs and vessels (18). However, hemothorax can occur without definitive reasons. Modi *et al* (19) reported that three cases of hemothorax occurred within one week after surgery in 27 cases of scoliosis with Duchenne muscular atrophy; however, computed tomography scans did not find any inappropriate positioning of the fixation apparatus. Modi hypothesized that the hemothorax may have been caused by the cortex penetrating the lateral or front side of the vertebrae during tapping or placing of the screws. In the present study, no abnormalities of the screw paths or injuries of the pulmonary tissues or vessels were found; therefore the incidence of hemothorax may be interpreted using the theory by Modi.

Posterior one-stage vertebral column resection has been increasingly adapted by spine surgeons (20). The greatest advantage of this surgery is that the surgeon is able to observe the spinal cord directly to decrease the risks of spinal injuries, and reduce the duration of surgery at the same time. Titanium mesh is a good choice for reconstruction of the spine, due to the varieties of shape, length and diameter that are available. The most common complications of titanium mesh are subsidence and shift (21). Six cases of mild subsidence occurred in the present study, which did not require revision. Subsidence of the replaced spinal segment was associated with increased duration of surgery, intraoperative bleeding volume and total blood transfusion volume. No available explanation for this connection was identified in the literature. However, X-rays showed that when the upper or lower end plates were partially resected, the exposure and bearing loss of the spongy bone could cause subsidence of the replaced spinal segment. This leads to the conclusion that the increased duration of surgery, intraoperative bleeding volume and total blood transfusion volume may indicate the spinal segment that is replaced during the surgery, and in such surgeries the end plate cannot be preserved well due to unclear visibility. However, once the prosthesis is fixed in position by the vertebral pedicle screws, it should not sink any further, and so should not lead to severe local kyphosis. Therefore, it is important to increase the extraversion angle of the screws to stop subsidence of the titanium mesh, which is a key process to avoid this type of complication. Artificial vertebral bodies were used in two cases without subsidence. They are expandable and easy to place during the surgery, and the risks of spinal cord injury are decreased; thus, from this point of view, they exhibits greater advantages than the mesh cage.

In addition, increased drainage volume caused by the allergic reactions to the allograft bone occurred in one case. Great attention was paid to this complication and it was considered to be associated with the immunogenicity of the allograft bone. In certain previous cases, removal of the allograft bone was necessary, and continuous drainage and antibiotic treatment were recommended subsequently (22); infection did not occur in these cases. However, in the present study, the drainage volume increased for 4 days after surgery and began to decrease gradually from postoperative day 5. Therefore, it was decided to retain the drainage and use antibiotics, to ensure that no infection occurred. This indicates that whether or not it is beneficial to remove the allograft bone depends on the individual case.

One case of acute enteritis was observed in the present study, and it was concluded that this may have been caused by unclean food. The patient subsequently healed following administration of antibiotics orally for three days. Two preoperative paraplegia patients suffered sensory decrease, but remained at Frankel Grade B, and two cases survived for <6 months. However, these four patients were satisfied with the outcome of the surgeries in terms of significant pain relief and improved life quality; therefore, the surgery indicators should also be individualized.

Univariate instead of multivariate analyses were used in the present study, due to the small number of patients recruited. This may have increased data deviations and influenced the accuracy of the result. Thus, further study and the collection of more data are required to improve the quality of the study. In conclusion, the present study demonstrates that the majority of the complications were minor and did not affect the prognosis of the patients. Active prevention is necessary to reduce the incidence of complications, in particular major ones.

## Figures and Tables

**Table I:** Classification and types of complications.

Classification	Complication	n
Intraoperative		
Major	–	0
Minor	Cerebral-spinal leakage	2
Early postoperative		
Major	Infective shock	1
Minor	Acute liver dysfunction and renal failure	1
	Drainage tube retention	1
	Late tracheal extubation	5
	Transient thrombocytopenia	10
	Hemothorax	2
	Pneumothorax	1
	Acute enteritis	1
	Transient cardiac ischemia	1
	Dissatisfied with the limited motor function improvement	2
Late postoperative		
Major	Regional recurrence	1
Minor	Hardware subsidence	6

**Table II:** Correlation of late tracheal extubation with intraoperative bleeding volume.

Tracheal extubation timing	Mean intraoperative bleeding volume, ml (range)
Late	7500 (3200–10750)
Normal	2000 (1500–3200)

Z=−2.367, P=0.018.

**Table III:** Correlation of late tracheal extubation with preoperative FVC and FEV1%.

Parameter	FVC	FEV1%
Late tracheal extubation	59.00±12.35	61.65±14.54
Normal tracheal extubation	80.26±14.20	80.91±14.21
t-value	2.864	2.563
P-value	0.007	0.015

FVC, forced vital capacity; FEV1%, forced expiratory volume in 1 sec.

**Table IV:** Correlation of replaced spinal segment subsidence with duration of surgery, intraoperative bleeding volume and total blood transfusion volume.

Parameter	Surgery duration, min (range)	Intraoperative bleeding volume, ml (range)	Total blood transfusion volume, ml (range)
With subsidence	380 (281.3–418.8)	6600 (2575–9550)	6700 (3250–9950)
Without subsidence	295 (245.0–335.0)	2000 (1300–3000)	2400 (1200–3600)
Z	−2.158	−2.895	−3.295
P-value	0.031	0.004	0.001

**Table V:** Correlation of thrombocytopenia with duration of surgery and total blood transfusion volume.

Parameter	Duration of surgery, min (range)	Total blood transfusion volume, ml (range)
Thrombocytopenia	380.0 (282.5–436.3)	3700 (2800–6800)
No thrombocytopenia	290.0 (242.5–330)	2400 (1200–3800)
Z	−2.719	−2.273
P-value	0.007	0.023
